# 

**Published:** 1916-09

**Authors:** 


70th ANNIVERSARY YEAR

### THE DENTAL REGISTER

Edited by Dr NELVILLE S. HOFF
ANN ARBOR, MICH.

VOL. LXX SEPTEMBER, 1916 NO. 9

LIBRARY
OCT. -2 1916
SURGEON GENERAL'S OFFICE

THE SAMUEL A. CROCKER CO., Publishers
OHIO DENTAL AND SURGICAL DEPOT
CONRAD BLDG., 18 & 20 W. Seventh St., CINCINNATI, O.

PRICE, ONE DOLLAR A YEAR

Entered at the Post-Office at Cincinnati, O., as second-class mail matter

